# Is Survival After Out-of-Hospital Cardiac Arrests Worse During Days of National Academic Meetings in Japan? A Population-Based Study

**DOI:** 10.2188/jea.JE20150100

**Published:** 2016-03-05

**Authors:** Tetsuhisa Kitamura, Kosuke Kiyohara, Tasuku Matsuyama, Toshihiro Hatakeyama, Tomonari Shimamoto, Junichi Izawa, Chika Nishiyama, Taku Iwami

**Affiliations:** 1 Division of Environmental Medicine and Population Sciences, Department of Social and Environmental Medicine, Graduate School of Medicine, Osaka University, Suita, Osaka, Japan; 2 Department of Public Health, Tokyo Women’s Medical University, Tokyo, Japan; 3 Department of Emergency Medicine, Kyoto Prefectural University of Medicine, Kyoto, Japan; 4 Kyoto University Health Service, Kyoto, Japan; 5 Department of Critical Care Nursing, Graduate School of Medicine and School of Health Sciences, Kyoto University, Kyoto, Japan

**Keywords:** out-of-hospital cardiac arrest, national academic meeting, outcome, cardiopulmonary resuscitation

## Abstract

**Background:**

Outcomes after out-of-hospital cardiac arrests (OHCAs) might be worse during academic meetings because many medical professionals attend them.

**Methods:**

This nationwide population-based observation of all consecutively enrolled Japanese adult OHCA patients with resuscitation attempts from 2005 to 2012. The primary outcome was 1-month survival with a neurologically favorable outcome. Calendar days at three national meetings (Japanese Society of Intensive Care Medicine, Japanese Association for Acute Medicine, and Japanese Circulation Society) were obtained for each year during the study period, because medical professionals who belong to these academic societies play an important role in treating OHCA patients after hospital admission, and we identified two groups: the exposure group included OHCAs that occurred on meeting days, and the control group included OHCAs that occurred on the same days of the week 1 week before and after meetings. Multiple logistic regression analysis was used to adjust for confounding variables.

**Results:**

A total of 20 143 OHCAs that occurred during meeting days and 38 860 OHCAs that occurred during non-meeting days were eligible for our analyses. The proportion of patients with favorable neurologic outcomes after whole arrests did not differ during meeting and non-meeting days (1.6% [324/20 143] vs 1.5% [596/38 855]; adjusted odds ratio 1.02; 95% confidence interval, 0.88–1.19). Regarding bystander-witnessed ventricular fibrillation arrests of cardiac origin, the proportion of patients with favorable neurologic outcomes also did not differ between the groups.

**Conclusions:**

In this population, there were no significant differences in outcomes after OHCAs that occurred during national meetings of professional organizations related to OHCA care and those that occurred during non-meeting days.

## INTRODUCTION

A total of approximately 120 000 out-of-hospital cardiac arrests (OHCAs) occur annually in Japan.^1^ Through the development of a public-access defibrillation system and dissemination of cardiopulmonary resuscitation (CPR) by bystanders,^2^^–^^5^ the outcomes after OHCA of cardiac origin among adults has been improving in communities, but outcomes are still poor, and the assessment of factors associated with improved survival after OHCA remains important in resuscitation science.^6^^–^^9^

Recently, some papers have suggested that survival rates after OHCA during nights or weekends were lower than during daytime and weekdays.^10^^–^^14^ One possible explanation for this phenomenon is poor in-hospital performances. Surprisingly, a report from the United States demonstrated that high-risk patients with cardiac arrest hospitalized in teaching hospitals had a lower 30-day mortality when admitted during dates of national cardiology meetings, when medical staffing in hospitals would be lower than during non-meetings days.^15^ However, no prior clinical studies have assessed the relationship between survival after OHCAs and the days of national academic meetings related to cardiology or resuscitation.

The All-Japan Utstein Registry is a large population-based observation of OHCAs that has been ongoing since January 2005.^1^^,^^2^ Using this database, we evaluated differences in 1-month survival with favorable neurologic outcome after adult OHCAs during national meeting and non-meeting days in Japan. Our hypothesis was that favorable neurologic outcome after OHCAs would be worse during days of national meetings because many medical professionals attend them.

## MATERIALS AND METHODS

### Study design and settings

The All-Japan Utstein registry of the Fire and Disaster Management Agency (FDMA) is a nationwide population-based OHCA registry based on Utstein-style data collection.^16^^,^^17^ This study enrolled all adult patients aged ≥18 years old who suffered an OHCA before emergency medical services (EMS) arrival, were resuscitated by bystanders or EMS personnel, and were transported to medical institutions between January 1, 2005, to December 31, 2012. The research protocol was approved by the Ethics Committee of Osaka University Graduate School of Medicine, and the requirement of informed consent was waived in accordance with the Japanese ethics guidelines for epidemiological studies. Details of the All-Japan Utstein registry, such as data collection and quality control, and the Japanese EMS system were previously described.^2^^,^^18^

Cardiac arrest was presumed as the cessation of cardiac mechanical activity, as confirmed by the absence of circulation signs.^16^^,^^17^ Cardiac arrest in the Utstein-style data was defined to be of cardiac origin unless it was caused by cerebrovascular diseases, respiratory diseases, malignant tumors, or external causes (including asphyxia, drug overdose, drowning, hanging, trauma, or any other non-cardiac cause). Diagnoses of cardiac or non-cardiac origin were made clinically by the corresponding physician in charge of the EMS personnel.

Living wills or do-not-resuscitate orders are generally not accepted in Japan, and EMS providers are not legally approved to terminate resuscitation in prehospital settings. Therefore, most OHCA patients treated by EMS personnel were transported to medical institutions and registered in the database. This registry excluded cases with definitive signs of death on EMS arrival.

### EMS systems in Japan

Japan had a population of approximately 127 million in 2005. EMS is provided by regional governments through fire departments. There were 752 fire departments with respective dispatch centers in 2012.^1^ An ambulance usually has three emergency providers as a crew, including at least one emergency life-saving technician (ELST), who are trained to insert an intravenous line, place an adjunct airway, and operate a semi-automated external defibrillator. Specially-trained ELSTs have also been able to insert an endotracheal tube and administer intravenous epinephrine since April 2006.^1^ Public-access defibrillation has been legally permitted since July 2004 in Japan. According to the Japanese CPR guidelines, EMS providers conduct CPR for OHCA patients.^6^

### Data collection and quality control

Based on the Utstein-style guidelines for reporting OHCA,^16^^,^^17^ data were collected using a specific form, which included details on age, sex, witness status, first recorded cardiac rhythm, time course of resuscitation (call received, ambulance arrival on the scene, contact with patient, CPR initiation, EMS defibrillation, and hospital arrival), bystander-initiated CPR, public-access automated external defibrillator (AED) use, intravenous epinephrine, and advanced airway management, as well as prehospital return of spontaneous circulation (ROSC), 1-month survival, and neurologic outcome 1 month after OHCA occurrence. The first documented rhythm was recorded and diagnosed by the EMS personnel with semi-automated defibrillators at the scene.

When laypersons delivered shocks using a public-access AED, the victims’ first documented rhythm was regarded as ventricular fibrillation (VF). Both bystander-initiated chest compression-only CPR and conventional CPR with chest compressions and rescue breathing were considered bystander CPR. The time of collapse and initiation of bystander CPR was obtained by EMS personnel who interviewed the bystander before leaving the scene. Neurologic status of survivors was evaluated 1 month after the event by the EMS personnel in charge.

The data sheet was filled out by EMS personnel with the physicians in charge of OHCA patients, and data were entered into the registry system on the FDMA database server. They were logically checked by the computer system and were confirmed by the working group. If the data form was not complete, the FDMA returned it to the respective fire station for completion.

### Key group definition

In this study, we focused on three national academic meetings (the Japanese Circulation Society [JCS],^19^ the Japanese Association for Acute Medicine [JAAM],^20^ and the Japanese Society of Intensive Care Medicine [JSICM]^21^), because we considered that medical professionals, such as doctors, nurses, and medical engineers, who belong to these academic societies play important roles in treating OHCA patients after hospital admission. The JCS, JAAM, and JSICM have a total of 25 863, 10 419, and 9402 members,^22^ respectively, and their national meetings are usually held for 3 consecutive days every March, October, and February, respectively. As an exception, the 2011 JCS meeting was held for 2 consecutive days in August, due to the Great East Japan Earthquake, which occurred in March 2011. Calendar days at these meetings were obtained for each year during the study period. For our analyses, we identified two groups: the exposure group, which included OHCAs that occurred on meeting days, and the control group, which included OHCAs that occurred during the same calendar days in the week before and after meetings.

### Main outcome measures

Neurologic outcomes were assessed using the Glasgow-Pittsburgh Cerebral Performance Category (CPC) scale and were categorized as 1: good performance, 2: moderate disability, 3: severe cerebral disability, 4: coma/vegetative state, and 5: death. The primary outcome measure was 1-month survival with a favorable neurologic outcome, defined as CPC category 1 or 2.^16^^,^^17^ The secondary outcome measure was 1-month survival.

### Statistical analysis

Characteristics of patients and prehospital care among eligible OHCA patients were compared between the exposure and control groups using Student’s *t*-test and χ^2^ test for categorical variables. Multivariable analysis with logistic regression models was used to compare the differences in outcomes between the two groups for 1-month survival and 1-month survival with a favorable neurologic outcome; adjusted odds ratios (ORs) and their 95% confidence intervals (CIs) were calculated. As potential prehospital confounders, factors that were biologically essential and considered to be associated with clinical outcomes were included in the multivariable analyses. These variables were age (adults aged 18–39 years, those aged 40–64 years old, or elderly aged ≥65 years), sex (men or women), origin of arrests (cardiac or non-cardiac), type of bystander witness (none, family member, or other), first documented rhythm (VF or non-VF), type of bystander-initiated CPR (none, compression-only CPR, or conventional CPR), public-access AED use (yes or no), intravenous fluid (yes or no), epinephrine (yes or no), advanced airway management (yes or no), time interval from call to hospital arrival (in 1-minute increments), and year of arrest (in 1-year increments). Chronological factors, namely the day of week (weekday or weekend) and time of day (on-duty hours [8:00 am to 4:59 pm] or off-duty hours [5:00 pm to 7:59 am]), were also incorporated into the multivariable models. In addition, the adjusted ORs were calculated for each meeting. In this study, we focused particularly on bystander-witnessed VF arrests of cardiac origin, because these patients could be subject to in-hospital advanced treatments, including percutaneous coronary intervention (PCI),^23^^,^^24^ therapeutic hypothermia (TH),^25^^,^^26^ and extracorporeal CPR (ECPR).^27^^,^^28^

All of the tests were two-tailed, and a *P* value of <0.05 was considered statistically significant. Statistical analyses were implemented using the SPSS statistical package version 22.0J (IBM Corp., Armonk, NY, USA).

## RESULTS

During the study period, a total of 907 760 instances of adult OHCA were documented, resuscitation was attempted in 894 814 victims, and 819 900 victims had arrests before EMS arrival (Figure). After excluding victims having arrests during non-eligible days, those without information on bystander-initiated CPR and first documented rhythm, and those with bystander-initiated rescue breathing-only CPR, 59 003 subjects (20 143 in the exposure group and 38 860 in the control group) were eligible for our analyses.

**Figure.**
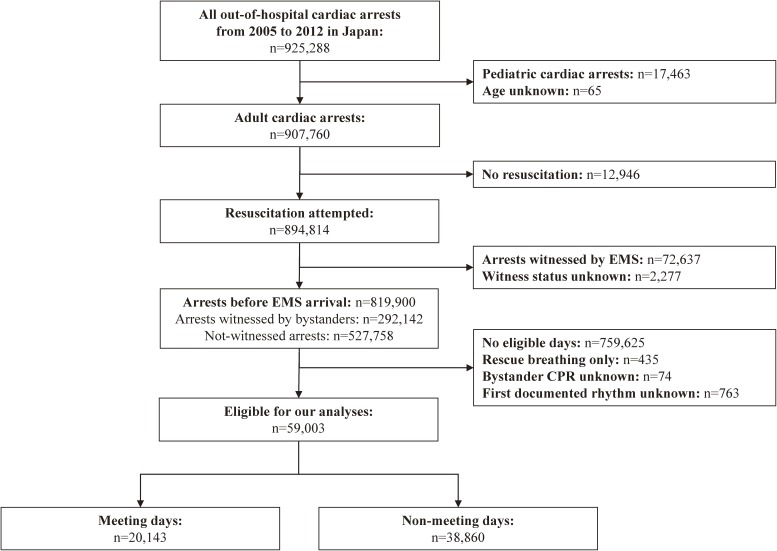
Study flow of pediatric out-of-hospital cardiac arrest cases with an abridged Utstein template from January 1, 2005 through December 31, 2012. EMS, emergency medical service; CPR, cardiopulmonary resuscitation.

The patient and EMS characteristics of OHCA events between academic meeting and non-meeting days are shown in Table 1. Among victims in both groups, the proportion of men was about 58%, and the mean age was 77 years. There were no differences between meeting and non-meeting days with regard to the etiology of arrests, type of witness status, first documented rhythm, type of bystander CPR, shocks by public-access AEDs, dispatcher instruction, and advanced life support offered, such as intravenous fluid, epinephrine administration, and advanced airway management by EMS personnel. The distribution of the day of week statistically differed between the two groups, but their proportions were almost the same. The EMS response time (call to contact with a patient) and hospital arrival time (call to hospital arrival) were almost the same between groups.

**Table 1.** Patient and EMS characteristics from out-of-hospital cardiac arrest during meeting and non-meeting days

	Meeting days (*n* = 20 143)	Non-meeting days (*n* = 38 860)	*P*
Men, *n* (%)	11 586 (57.5)	22 372 (57.6)	0.904
Age, years, median (IQR)	77 (65–85)	77 (65–85)	0.518
Age group, *n* (%)			0.379
18–39 years	919 (4.6)	1677 (4.3)	
40–64 years	3837 (19.0)	7440 (19.1)	
≥65 years	15 387 (76.4)	29 743 (76.5)	
Etiology, *n* (%)			0.504
Cardiac	11 436 (56.8)	22 174 (57.1)	
Non-cardiac	8707 (43.2)	16 686 (42.9)	
External cause	3565 (40.9)	6859 (41.1)	
Respiratory disease	1058 (12.2)	2152 (12.9)	
Cerebrovascular disease	861 (9.9)	1693 (10.1)	
Malignant tumor	650 (7.5)	1239 (7.4)	
Other	2573 (29.6)	4743 (28.4)	
Type of witness status, *n* (%)			0.089
None	13 226 (65.7)	25 242 (65.0)	
Arrests witnessed by bystanders	6917 (34.3)	13 618 (35.0)	
Family member	4384 (63.4)	8671 (63.7)	
Friend	271 (3.9)	535 (3.9)	
Colleague	235 (3.4)	459 (3.4)	
Passer-by	338 (4.9)	683 (5.0)	
Other	1689 (24.4)	3270 (24.0)	
First documented rhythm, *n* (%)			0.743
VF	1598 (7.9)	3033 (7.8)	
PEA	3979 (19.8)	7759 (20.0)	
Asystole	14 566 (72.3)	28 068 (72.2)	
Type of bystander CPR, *n* (%)			0.915
No CPR	11 615 (57.7)	22 470 (57.8)	
Chest compression-only CPR	6022 (29.9)	11 593 (29.8)	
Conventional CPR	2506 (12.4)	4797 (12.3)	
Shocks by public-access AEDs, *n* (%)	156 (0.8)	312 (0.8)	0.712
Dispatcher instruction, *n* (%)	9384 (46.6)	18 363 (47.3)	0.124
Intravenous fluid, *n* (%)	5079 (25.2)	9633 (24.8)	0.257
Epinephrine administration, *n* (%)	1643 (8.2)	3162 (8.1)	0.934
Advanced airway management, *n* (%)	8918 (44.3)	17 227 (44.3)	0.894
Day of week, *n* (%)			0.008
Weekdays	13 588 (67.5)	25 793 (66.4)	
Weekends	6555 (32.5)	13 067 (33.6)	
Time of day, *n* (%)			0.683
On-duty hours (8:00 am to 4:59 pm)	8519 (42.3)	16 503 (42.5)	
Off-duty hours (5:00 pm to 7:59 am)	11 624 (57.7)	22 357 (57.5)	
EMS resuscitation times, min, median (IQR)			
EMS response time (call to contact with a patient)	8 (6–10)	8 (6–10)	0.866
Hospital arrival time (call to hospital arrival)	30 (24–38)	30 (24–38)	0.669

AED, automated external defibrillator; CPR, cardiopulmonary resuscitation; EMS, emergency medical service; IQR, interquartile range; PEA, pulseless electrical activity; VF, ventricular fibrillation.

Table 2 shows the proportion of subjects with 1-month survival and with 1-month survival with a favorable neurologic outcome after whole arrests and bystander-witnessed VF arrest of cardiac origin arrests between meeting and non-meeting days. The proportion of subjects experiencing the primary and secondary outcomes after whole arrests did not differ markedly between meeting and non-meeting days (3.8% [774/20 143] versus 3.8% [1477/38 859] for 1-month survival [adjusted OR 1.01; 95% CI, 0.92–1.11]; and 1.6% [324/20 143] versus 1.5% [596/38 855] for 1-month survival with a favorable neurologic outcome [adjusted OR 1.02; 95% CI, 0.88–1.19]). As for bystander-witnessed VF arrests of cardiac origin, the proportion of subjects experiencing the primary and secondary outcomes after whole arrests did not differ between meeting and non-meeting days (28.9% [275/951] versus 26.5% [481/1814] for 1-month survival [adjusted OR 1.10; 95% CI, 0.92–1.33]; and 19.1% [182/951] versus 17.9% [324/1813] for 1-month survival with a favorable neurologic outcome [adjusted OR 1.04; 95% CI, 0.83–1.29]).

**Table 2.** Outcomes of out-of-hospital cardiac arrest during meeting and non-meeting days

	Meeting days	Non-meeting days	*P*
Whole arrests			
1-month survival, % (*n*/*N*)	3.8 (774/20 143)	3.8 (1477/38 859)	
Crude OR (95% CI)	1.01 (0.93–1.11)	Reference	0.802
Adjusted OR (95% CI)	1.01 (0.92–1.11)	Reference	0.799
Favorable neurologic outcome, % (*n*/*N*)	1.6 (324/20 142)	1.5 (596/38 855)	
Crude OR (95% CI)	1.05 (0.92–1.08)	Reference	0.488
Adjusted OR (95% CI)	1.02 (0.88–1.19)	Reference	0.757
Bystander-witnessed VF arrests with cardiac origin			
1-month survival, % (*n*/*N*)	28.9 (275/951)	26.5 (481/1814)	
Crude OR (95% CI)	1.12 (0.94–1.35)	Reference	0.179
Adjusted OR (95% CI)	1.10 (0.92–1.33)	Reference	0.297
Favorable neurologic outcome, % (*n*/*N*)	19.1 (182/951)	17.9 (324/1813)	
Crude OR (95% CI)	1.09 (0.89–1.33)	Reference	0.413
Adjusted OR (95% CI)	1.04 (0.83–1.29)	Reference	0.750

CI, confidence interval; OR, odds ratio; VF, ventricular fibrillation.

ORs were adjusted for origin of arrest, gender, age, type of witnessed status, first documented rhythm, public-access AED shocks, dispatcher instruction, type of bystander CPR, intravenous fluid, epinephrine, advanced airway management, hospital arrival time, day of week, time of day, and year.

A total of 155 1-month survival and 160 favorable neurologic outcome data points were missing.

Table 3 show outcomes of OHCAs during meeting and non-meeting days by the type of national meeting. Even after adjusting for potential confounding factors, there were no significant differences in the number of subjects with a favorable neurologic outcome after whole arrests during meeting and non-meeting days (1.5% [104/6843] versus 1.5% [215/13 893] for JCS meetings [adjusted OR 0.98; 95% CI, 0.76–1.26]; 1.9% [114/6156] versus 1.9% [224/12 098] for JAAM meetings [adjusted OR 1.03; 95% CI, 0.80–1.32]; and 1.5% [114/7728] versus 1.3% [193/14 983] for JSICM meetings [adjusted OR 1.09; 95% CI, 0.85–1.41]). Regarding VF arrests, the outcomes also did not differ between the groups, irrespective of the type of meeting.

**Table 3.** Outcomes of out-of-hospital cardiac arrest during meeting and non-meeting days by the type of national meeting

	Japanese Circulation Society		*P*	Japanese Association for Acute Medicine		*P*	Japanese Society of Intensive Care Medicine		*P*
		
	Meeting days	Non-meeting days		Meeting days	Non-meeting days		Meeting days	Non-meeting days	
Whole arrests									
1-month survival, % (*n*/*N*)	3.7 (250/6844)	3.7 (520/13 896)		4.1 (255/6156)	4.3 (520/12 098)		3.7 (289/7728)	3.5 (524/14 984)	
Crude OR (95% CI)	0.98 (0.84–1.14)	Reference	0.749	0.96 (0.83–1.12)	Reference	0.621	1.07 (0.93–1.24)	Reference	0.351
Adjusted OR (95% CI)	0.99 (0.84–1.16)	Reference	0.866	0.99 (0.84–1.16)	Reference	0.882	1.05 (0.90–1.23)	Reference	0.544
Favorable neurologic outcome, % (*n*/*N*)	1.5 (104/6843)	1.5 (215/13 893)		1.9 (114/6156)	1.9 (224/12 098)		1.5 (114/7728)	1.3 (193/14 983)	
Crude OR (95% CI)	0.98 (0.77–1.24)	Reference	0.879	1.00 (0.80–1.26)	Reference	0.999	1.15 (0.91–1.45)	Reference	0.248
Adjusted OR (95% CI)	0.98 (0.76–1.26)	Reference	0.858	1.03 (0.80–1.32)	Reference	0.814	1.09 (0.85–1.41)	Reference	0.491
Bystander-witnessed VF arrests with cardiac origin									
1-month survival, % (*n*/*N*)	27.5 (90/327)	26.6 (177/665)		30.3 (91/300)	28.7 (177/617)		28.1 (98/349)	25.7 (168/654)	
Crude OR (95% CI)	1.05 (0.78–1.41)	Reference	0.762	1.08 (0.80–1.46)	Reference	0.607	1.13 (0.84–1.51)	Reference	0.414
Adjusted OR (95% CI)	1.08 (0.78–1.48)	Reference	0.650	1.06 (0.77–1.47)	Reference	0.724	1.04 (0.77–1.42)	Reference	0.793
Favorable neurologic outcome, % (*n*/*N*)	17.4 (57/327)	17.8 (118/664)		23.3 (70/300)	19.4 (120/617)		16.6 (58/349)	17.1 (112/654)	
Crude OR (95% CI)	0.98 (0.69–1.38)	Reference	0.895	1.26 (0.90–1.76)	Reference	0.174	0.96 (0.68–1.37)	Reference	0.839
Adjusted OR (95% CI)	0.96 (0.66–1.41)	Reference	0.849	1.27 (0.88–1.82)	Reference	0.203	0.86 (0.59–1.26)	Reference	0.440

CI, confidence interval; OR, odds ratio; VF, ventricular fibrillation.

ORs are adjusted for origin of arrest, gender, age, public-access automated external defibrillator shocks, dispatcher instruction, type of bystander cardiopulmonary resuscitation, intravenous fluid, epinephrine, advanced airway management, hospital arrival time, on duty time/off duty time, weekday/weekend, and year.

## DISCUSSION

Contrary to our hypothesis, there were no significant differences in 1-month survival and favorable neurologic outcome after adult OHCAs in the nationwide OHCA registry in Japan during national academic meeting and non-meeting days. Notably, this result was not consistent with findings from a previous paper.^15^ Since evidence concerning the relationship between outcomes after OHCAs and the period of national meetings is scarce, this study is genuinely unique and may provide helpful information for improving resuscitation practice.

Although Jena and colleagues observed a lower 30-day mortality among patients with cardiac arrest admitted to major teaching hospitals during the dates of two national cardiology meetings (those of the American Heart Association and the American College of Cardiology) in the United States,^15^ we did not find similar results from our registry. Jena et al speculated the following reasons for their findings: (1) selective declines in cardiologist staffing (eg, the composition of physicians who remain to treat hospitalized patients), (2) declines in intensity of care during meetings (eg, reluctance to perform interventions for patients whose primary cardiologist is unavailable, or reluctance of cardiologists to intervene in high-risk patients without adequate back-up), and (3) declines in the volume of less urgent cardiovascular hospitalizations during meeting periods (eg, physicians could focus greater attention on the remaining high-risk patients). These three possibilities may occur during Japanese national meetings, but the resulting differences between meeting and non-meeting days might be small in the medical systems of Japan.

There are other possibilities to explain our results. First, resuscitation specialists in Japan might maintain their clinical skills with mutual cooperation and continuous effort. Second, medical professionals who are engaged in emergency care might participate in regional meetings in order to obtain credit as a specialist, because these meetings are held in areas relatively near their hospitals and have shorter duration than larger meetings (generally a day or two, compared to larger three-day meetings). The reasons addressing the association between exposure factors and the results observed here were insufficient; therefore, the lack of differences in OHCA outcomes between meeting and non-meeting days requires further study.

In this study, we focused especially on bystander-witnessed VF arrests of cardiac origin. The proportion of subjects with a favorable neurologic outcome among VF patients is well-known to be higher than that among other OHCA patients,^2^^,^^3^^,^^29^ and in-hospital advanced treatments would be provided for these patients by specialists who belong to academic societies related to cardiology, such as JCS, JAAM, and JSICM. Indeed, the preceding reports underscored that PCI, TH, and ECPR contributed to improving the outcomes of VF patients.^23^^–^^28^ Jena and colleagues also showed that high-risk patients with acute myocardial infarction (AMI) admitted to teaching hospitals during meetings were less likely to receive PCI.^15^ Therefore, implementation rates of these advanced treatments for OHCA patients might be reduced during Japanese national meeting days. However, considering that the proportion of subjects with favorable neurologic outcome of bystander-witnessed VF arrests of cardiac origin did not differ during meetings and non-meeting days for each professional society, we speculate that advanced treatments do not increase or decrease during meeting days. This study, which was based on the Utstein-style data, did not obtain information on in-hospital advanced treatments for OHCA patients, so further studies to assess differences in utilization of advanced treatments during meeting and non-meeting days are needed.

On the other hand, recent reports investigating the effect of the time of day or the day of the week on outcomes among adult^10^^–^^12^ and pediatric^14^ patients with OHCA or in-hospital cardiac arrest^13^ showed that survival after OHCA was significantly lower during nights and weekends than during daytime and weekdays. On nights and weekends, the number of staff, such as physicians and nurses, was generally smaller than during daytime and weekdays. In addition, emergency physicians were less effective when performing manual and cognitive tests while working night shifts than when working day shifts.^13^ Medical resource allocation during nights and weekends, when in-hospital performance deteriorates, is an important issue in OHCA treatment.^30^ Although we did not find a significant relationship between OHCA outcomes and attendance at national meetings in this study, staff composition and rotation during meeting days should also be assessed in Japan.

From our results, it is “acceptable” that medical professionals who engage in OHCA treatments in Japan attend national academic meetings, since these professionals may learn about the latest findings of resuscitation science by attending. However, epidemiological studies assessing the effects of meeting periods on OHCA outcomes in other countries are required to confirm whether or not our results are generalizable outside of Japan. In addition, detailed investigation of outcomes and procedures for patients admitted to hospitals during meetings and non-meeting days with AMI, acute heart failure, sepsis, or trauma should be assessed, and these accumulated data may well provide further insights for improving the quality of cardiology, emergency medicine, and intensive care medicine.

The present study has several limitations. First, this study did not obtain detailed information on medical professionals attending national meetings and non-participants remaining in hospitals. In addition, we focused on OHCAs that occurred during three representative national meetings for the purpose of simplifying our research question, but the relationship between survival after acute cardiovascular diseases, including OHCAs, and both regional and international meeting periods should be assessed in the future. Second, we did not address the influence of the type of hospital providing the data. For instance, Jena showed that no mortality or utilization differences existed for patients in nonteaching hospitals.^15^ Therefore, our results might have differed if we had information regarding the type or volume of hospitals. Third, unmeasured confounding factors might have influenced the association between OHCAs that occurred during meeting days and the outcomes measured here. Fourth, as with all epidemiologic studies, the integrity and validity of the data, as well as ascertainment bias, are potential limitations of our study. However, the use of uniform data collection based on Utstein-style guidelines for reporting cardiac arrest, the large sample size, and the population-based design should minimize these potential sources of bias.

### Conclusions

In this population, there were no significant differences in outcomes after adult OHCA between meeting and non-meeting days. Other epidemiological studies that assess the effects of meeting periods on OHCA outcomes are required to confirm our results.
